# Evaluation of effective quantum yields of photosystem II for CO_2_ leakage monitoring in carbon capture and storage sites

**DOI:** 10.7717/peerj.10652

**Published:** 2021-01-28

**Authors:** Wenmei He, Gayoung Yoo, Youngryel Ryu

**Affiliations:** 1 Department of Applied Environmental Science, Kyunghee University, Yongin-si, South Korea; 2 Department of Landscape Architecture and Rural System Engineering, Seoul National University, Seoul, South Korea

**Keywords:** Carbon capture and storage, CO_2_ leakage, Chlorophyll fluorescence, Hyperspectral sensing, Index evaluation score

## Abstract

Vegetation monitoring can be used to detect CO_2_ leakage in carbon capture and storage (CCS) sites because it can monitor a large area at a relatively low cost. However, a rapidly responsive, sensitive, and cost-effective plant parameters must be suggested for vegetation monitoring to be practically utilized as a CCS management strategy. To screen the proper plant parameters for leakage monitoring, a greenhouse experiment was conducted by exposing kale (*Brassica oleracea* var. viridis), a sensitive plant, to 10%, 20%, and 40% soil CO_2_ concentrations. Water and water with CO_2_ stress treatments were also introduced to examine the parameters differentiating CO_2_ stress from water stresses. We tested the hypothesis that chlorophyl fluorescence parameters would be early and sensitive indicator to detect CO_2_ leakage. The results showed that the fluorescence parameters of effective quantum yield of photosystem II (Y(II)), detected the difference between CO_2_ treatments and control earlier than any other parameters, such as chlorophyl content, hyperspectral vegetation indices, and biomass. For systematic comparison among many parameters, we proposed an indicator evaluation score (IES) method based on four categories: CO_2_ specificity, early detection, field applicability, and cost. The IES results showed that fluorescence parameters (Y(II)) had the highest IES scores, and the parameters from spectral sensors (380–800 nm wavelength) had the second highest values. We suggest the IES system as a useful tool for evaluating new parameters in vegetation monitoring.

## Introduction

Carbon capture and storage (CCS) technology, which has been developed in many countries over the last two decades, is a potentially useful and promising way to reduce anthropogenic CO_2_ emissions (IPCC, 2005, Pachauri & Meyer, 2014; European Commission & European Communities, 2011; Cartier, 2020). CO_2_ leakage probability is low when CCS site selection, characterization, and project design are appropriately undertaken. Moreover, the guidance documents from the Storage Directive (European Commission & European Communities, 2011) provide a legislative framework for appropriate project design to ensure permanent and safe CO_2_ storage (Pruess, 2011; Chen et al., 2017). Nevertheless, potential CO_2_ leakages from deep storage sites through injection wells, abandoned wells, geological faults, or fractures should not be ignored (IPCC, 2005; Jones et al., 2014; Jiang et al., 2015). Consequently, sensitive and effective monitoring of CO_2_ leakage is essential for safe and successful CCS applications (IPCC, 2005; Pearce et al., 2014; Vrålstad et al., 2018).

Conventionally, many buried CO_2_ sensors would be used to detect CO_2_ leakage because the location of leakage is unpredictable. Therefore, it is costly to monitor a large area (Noble et al., 2012). As an alternative, vegetation monitoring has been suggested to detect leakage across a large area in a cost-effective manner (Vodnik et al., 2002; Pfanz et al., 2004; Patil, 2012; Noble et al., 2012). Plants grown near a leak are known to be negatively affected by increased CO_2_ concentrations in the soil. Therefore, considerable changes in leaf color, chlorophyl content, plant height, and biomass can be observed within a few days to months after leakage (Beaubien et al., 2008; Krüger et al., 2011; Sharma et al., 2014; Lake et al., 2016a). Among those CO_2_-affected plant parameters, chlorophyl content was suggested to be a more appropriate parameter than biomass because it can be measured using nondestructive methods such as spectral sensors. However, the change in chlorophyl content due to elevated soil CO_2_ is generally observed 7–15 days after the initiation of CO_2_ leakage (Patil, 2012; Zhang et al., 2016; He et al., 2019a), showing that it is not an early indicator. Moreover, chlorophyl content change could not differentiate CO_2_ stress from other environmental stresses in the field (Sharma et al., 2014). Hyperspectral sensors were introduced in this field of study because they can detect various symptoms of plant stress by measuring an extensive range of reflectance signatures. CO_2_ and water stress were detected in the visible to near-infrared regions (VNIR: 380–800 nm) with normalized difference vegetation index (NDVI) and infrared regions (SWIR: 800–1,400 nm) with normalized difference water index (NDWI), respectively (Male et al., 2010; Lakkaraju et al., 2010; Wimmer et al., 2011). In the USA, Male et al. (2010) demonstrated that spectrometer payloads aboard unmanned aerial vehicles were successful in detecting CO_2_ leakage in the large artificial gassing site of the Zero Emissions Research and Technology (ZERT) project. In the UK, Jiang et al. (2015) detected CO_2_ leakage using a spectroradiometer in an artificial soil gassing and response detection (ASGARD) site, and in Australia, Feitz et al. (2014) observed a change in plant spectral responses under CO_2_ stress at the Ginninderra experiment station. However, these studies reported that the change in hyperspectral parameters was evident within 7–14 days. This indicates that the parameters from spectral sensing could not detect CO_2_ leakage in the early stages. In order to overcome the low sensitivity of these parameters, photosynthetic process-based parameters were tested to determine whether they are more sensitive to CO_2_ leakage detection. The photosynthetic rate, stomatal conductance, and transpiration rate were reported to decrease within 1–4 days in soil with high CO_2_ (>40%) conditions (Lake et al., 2016b; Zhang et al., 2016; He et al., 2019b). Consequently, they are potential early indicators for detecting CO_2_ leakage. However, the measurements of these parameters are generally laborious and require time-consuming procedures (Lake et al., 2016b; Zhang et al., 2016).

Recently, chlorophyl fluorescence parameters derived from photochemistry have been suggested as sensitive indicators for the early detection of plant responses to environmental stresses such as drought, salinity, diseases, and extreme temperature (Jiang et al., 2006; Roháček, Soukupová & Barták, 2008; Živčák et al., 2008; Li et al., 2013). Empirical fluorescence parameters, such as maximum quantum yield of photosystem II (Fv/Fm) and non-photochemical quenching (NPQ), have been proposed as the most sensitive indicators to detect plant responses to stress. However, Fv/Fm and NPQ measurement should be conducted in leaves adapted in the dark for at least 30 min before measurements are taken (Baker, 2008; Narayan, Misra & Singh, 2012; Murchie & Lawson, 2013). Thus, Fv/Fm and NPQ are not suitable for the rapid detection of plant stresses, especially under field conditions. Recently, advancements in fluorometer technology have supported the measurement of these parameters under natural light conditions, that is Y(II), which gives the proportion of absorbed light that is used in photosystem II photochemistry. Y(II) has been suggested as valuable indicators of plant stress because it does not have the limitation of leaf dark adaptation (Murchie & Lawson, 2013). Although the accurate measurement of Y(II) is still challenging under natural conditions, a new advanced monitoring pulse-amplitude-modulation fluorometer (MONI-PAM) would be a reliable tool for measuring Y(II) during daylight periods (Porcar-Castell et al., 2008). Y(II) was reported to detect plant drought stress after 1–2 days of treatment in a laboratory study (Li et al., 2013). Although the application of chlorophyl fluorescence parameters has rarely been reported in CO_2_ leakage monitoring, we firstly hypothesize that the fluorescence parameters of Y(II) derived from photochemical processes could be a useful indicator to quickly detect CO_2_ leakage, because the photosynthesis process is known to be affected by elevated soil CO_2_ (Zhang et al., 2015).

However, early detection is only one of the criteria to be a good indicator. The best parameter should not only detect CO_2_ leakage early but also discriminate CO_2_ stress from other environmental stresses, such as drought or heat. At the same time, it might cover large areas of monitoring at a relatively low cost (Male et al., 2010; Noble et al., 2012; Murchie & Lawson, 2013). To select the optimal indicator for CO_2_ leakage monitoring, there is a need to have a systematic comparison among suggested plant indicators for detecting CO_2_ leakage.

This study is composed of two parts. In the first part, we conducted a greenhouse experiment to simulate CO_2_ leakages. The aims were to (1) examine the possibility of chlorophyl fluorescence parameters for detecting CO_2_ leakage; (2) identify the parameters that could be used to distinguish CO_2_ and water stresses. In the second part, based on the results from our experiment and by synthesizing extensive findings from other studies, we developed a framework to evaluate indicators to provide the guideline for selecting the optimal parameter for CO_2_ leakage monitoring (Male et al., 2010; Noble et al., 2012; Lake et al., 2013)

## Materials and Methods

### Soils, plants, and injection pot

The greenhouse experiment was conducted at the campus of Kyung Hee University, South Korea (37°14′24″ N, 127°5′2″ E) (He et al., 2019b). The soils used in this experiment were a 1:1 (v/v) mixture of potting and mineral soils. Commercial potting soil was procured from the Korea Association of Seedbed Media in South Korea. Mineral soil was collected from the Environmental Impact Evaluation Test Facility in South Korea (36°57′44″ N, 127°28′3″ E). This facility is an artificial gassing site that was established to develop environmental management techniques for soil, groundwater, atmosphere, and ecosystems in CO_2_ storage sites. The basic physicochemical properties are summarized in Table 1. Three-leaf stage kale (*Brassica oleracea* var. viridis), purchased from West Suwon Agricultural Products Inc. (Suwon, South Korea), was prepared for the experiment. Kale was selected as our testing plant for the following reasons: (1) it is known to be sensitive to environmental stress (Tang et al., 2014), and (2) the leaves are sufficiently large to be covered by the chambers of measurement devices.

**Table 1:** Physicochemical properties of potting and mineral soils used for the experiment.

Soil	pH	TN	TC	Composition (%)					
		-g kg^−1^ soil-		Zeolite	Pearlite	Vermiculite	Coco peat	Peat moss	Other
Potting	6.5	6.3	390.3	4.1	7.3	6.6	68.0	14.7	0.3
				**Clay**	**Silt**	**Sand**			
Mineral	5.3	0.5	3.7	8	25	67			

**Figure 1:**
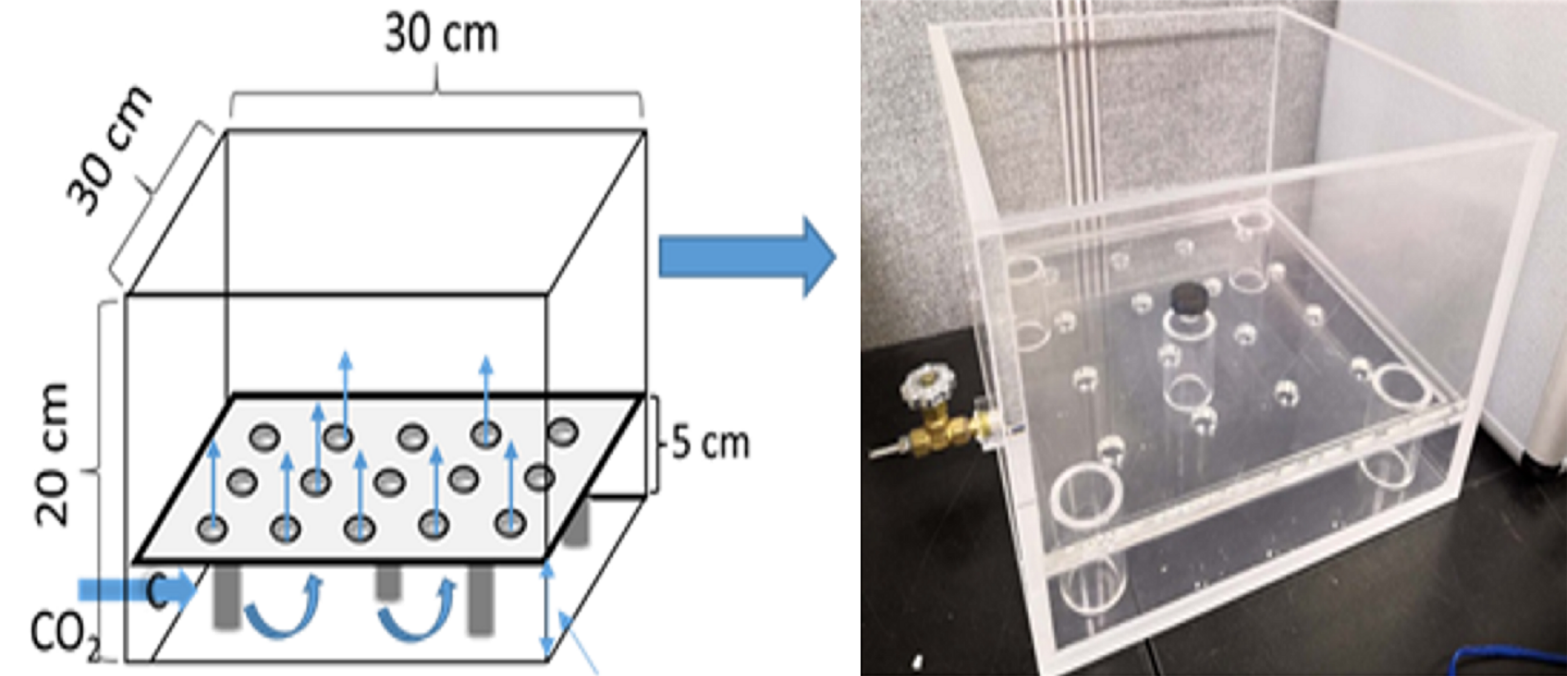
The design diagram and photo of the injection pot.

A specially designed acrylic pot consisted of an upper soil chamber (15 × 30 × 30 cm, i.e., height × length × width, respectively) and bottom injection space (5 × 30 × 30 cm, i.e., height × length × width, respectively) (Fig. 1). A clapboard between the soil and gassing chambers had 16 holes drilled into it and was covered with mesh (pore diameter: 250 µm) for optimal gas diffusion. The soil chamber was filled with 8 kg of soil mixture to a depth of 15 cm. Seedlings were transplanted into the soil chamber with 12 plants per pot on September 13, 2018.

### Treatment design

Three CO_2_ treatments included 10%, 20%, and 40% soil CO_2_ concentrations (labeled 10% CO_2_, 20% CO_2_, and 40% CO_2_, respectively). Pure CO_2_ gas was continuously injected into the gassing pots from October 10 to 20, 2018. The soil CO_2_ concentrations were adjusted by controlling the injection flow rate using a flow meter. A control plot was prepared without any CO_2_ injection. Soil water contents in the CO_2_ treatments and control were maintained at 55–65% of soil water-holding capacity (WHC) by watering daily at 500–800 ml per pot during the experimental period. To differentiate the plant responses to CO_2_ stress to those with water stress, we prepared a water stress treatment (WATER) and water stress with 40% CO_2_ treatment (WATER + CO_2_). Soil water contents in the WATER and WATER + CO_2_ treatments were maintained at 25–35% of WHC by watering daily at 100–150 ml per pot. These soil water contents were considered to be a mild water stress condition for kale (Li et al., 2013; Xavier et al., 2017). Each treatment included three replicated pots. During the experimental period, the ambient temperature was 19 ± 3 °C during the day and 6 ± 3 °C at night. The greenhouse remained open and two fans were used to prevent ambient CO_2_ accumulation which might affect the plant leaf level CO_2_ concentration (He et al., 2019b).

### Measurement of soil CO_2_, O_2_ concentrations, and water content

The CO_2_ and O_2_ concentrations were measured daily using a portable GA5000 gas sensor (for CO_2_%) ranging from 1 to 100% (volume with ±1% accuracy) (Geotechnical Instruments UK Limited, Coventry, UK) at 10 cm depth. Soil water content (% volumetric basis) was measured daily using a Decagon 5TM soil moisture sensor (Decagon Devices, Inc., Washington, DC, USA).

### Plant measurements

The overall visual changes in the plants in each treatment were recorded by taking photographs every 2 days. Measurements of reflectance signature, photosynthetic parameters, chlorophyl content, and chlorophyl fluorescence parameters were measured on the third to the fifth fully expanded leaves. These selected leaves were in a similar developmental stage, which would minimize variation due to different physiological ages of the leaves (Mendelssohn, McKee & Kong, 2001). Plant measurements were conducted between 10:00 am and 16:30 pm on October 10, 13, 15, and 18 (i.e., 0, 3, 5, and 8 days after experiment onset, respectively), 2018. The hyperspectral reflectance signatures from wavelengths 350 to 2500 nm (spectral resolution: 3 nm VNIR, 30 nm SWIR) were measured using ASD FieldSpec Pro (Malvern Panalytical Ltd. Cambridge, Malvern, UK). Leaf level measurements were conducted on 10 leaves in each pot. The spectral signatures were converted to readable reflectance values using the ViewSpecPro software (Vision 6.2; ASD, Inc., Falls Church, VA, USA). The vegetation indices, including the NDVI, photochemical reflectance index (PRI), and enhanced vegetation index (EVI), were calculated to monitor leaf pigment changes according to the equations in Table 2. The NDVI is a popular vegetation index, which has been shown to be strongly related to chlorophyl light interception (Tucker, 1979; Hatfield et al., 2008). On the other hand, the PRI is an indicator of changes in carotenoid pigments, which could also imply environmental stress (Gamon, Peñuelas & Field, 1992; Penuelas et al., 1997). The EVI is an alternative index to assess vegetation greenness and has also been used to detect CO_2_ leakage (Bateson et al., 2008). Furthermore, the NDWI and modified normalized difference water index (mNDWI) were correlated with the water content of plants (Xu, 2006; Male et al., 2010; Gautam et al., 2015), and were also calculated following the equations in Table 2.

**Table 2:** Equations used to calculate vegetation indices.

Indices	Formula	References
NDVI	(R_800_* – R_670_)/(R_800_ + R_670_)	Tucker (1979)
EVI	2.5 × ((R_800_ − R_670_)/(R_800_ + 6 × R_670_ − 7.5 × R_470_ + 1))	Huete et al. (2002)
PRI	(R_570_ − R_531_)/(R_531_ + R_570_)	Gamon, Peñuelas & Field (1992)
NDWI	(R_860_ − R_1,240_)/(R_860_ − R_1,240_)	Gao (1996)
mNDWI	(R_1,280_ − R_1,450_)/(R_1,280_ + R_1,450_)	Xu (2006)

**Note:**

* The R_470_, R_531_, R_570_, R_670_, R_800_, R_860_, R_1,240_, and R_1,450_ represent the reflectance values at 470, 531, 570, 670, 800, 860, 1,240, and 1,450 nm, respectively.

Chlorophyll content was measured for 10 leaves in every pot using a chlorophyl meter (SPAD 502plus; Konica Minolta, Tokyo, Japan). This is a well-known method for estimating leaf chlorophyl concentration (Süß et al., 2015). The measurement head of the SPAD emits light with peak wavelengths at 650 nm and 940 nm when it clips a leaf. Part of the light was absorbed by chlorophyl, while the transmittance light was measured by a photodiode detector to calculate the relative chlorophyl content automatically (Süß et al., 2015).

The conventional photosynthetic parameters (i.e., photosynthetic rate (Pn), stomatal conductance (Gs), and transpiration rate (Tr)) were measured in three to five leaves per pot using an infrared gas analyzer by portable devices, LI-6400 and LI-6800 (LI-COR, Lincoln, NE, USA). The measurements were made with a red-light source at 1,000 µmol m^–2^ s^–1^ photosynthetic photon flux density (PPFD), which was near the plant photosynthetic saturation point (Kalaji et al., 2018; Casanova-katny, Barták & Gutierrez, 2019).

Chlorophyll fluorescence was measured using the WinControl-3 controlled MONI-PAM Fluorometer system (Heinz-Walz, Eifeltrich, Germany). The MONI-PAM system comprised three emitter-detector heads (MONI-head/485). These units can measure three leaves simultaneously because each head is an independent fluorometer. The head can provide an actinic light pulse up to 1500 μmol m^−2^ s^−1^ PPFD (Porcar-Castell et al., 2008; Janka et al., 2015). The MONI-PAM device measured the maximal fluorescence yield (F_m_′), steady-state fluorescence yield (F_s_′), and photosynthetically active radiation (PAR) on six leaves in each pot. The fluorescence parameters of Y(II) was calculated to estimate the plant stress responses by the formula: Y(II) = (F_m_′ – F_s_′)/F_m_′ (Maxwell & Johnson, 2000; Murchie & Lawson, 2013; Janka et al., 2015). Y(II) was analyzed using a modified regression method by plotting the light response curve of Y(II) to PAR for each leaf (Sven, Mates & John, 2014; Casanova-katny, Barták & Gutierrez, 2019). The leaf level PAR values were adjusted by controlling the emitter-detector head to emit actinic light pulse incrementally (i.e., 100, 200, 300, 450, 650, 800, 1,200, and 1,500 µmol m^−2^ s^−1^). The Y(II) values was obtained from the curve at PAR = 1,000 μmol m^−2^ s^−1^.

After the injection stopped, plants were harvested and oven-dried at 70 °C for 3 days to measure biomass.

### Evaluation of plant parameters

The indicator evaluation score (IES) was developed to compare the efficiencies of the parameters for CO_2_ leakage monitoring. Based on extensive reviews, we set up four criteria for IES, including early detection, CO_2_ specificity, field applicability, and cost (Male et al., 2010; Patil, 2012; Al-Traboulsi et al., 2012; Noble et al., 2012; He et al., 2019a). The scores in each criterion, ranging from 1 to 5, were allocated for each parameter based on the evaluation standards in Table 3.

**Table 3:** Evaluation standards for the allocation of scores in the criteria.

Score	CRITERIA			
**Early detection**	**Initial day for observing plant changes (day)**		**Differentiate control and CO_2_ (≥10%) stress treatment**	
5	≤1		Y*	
4	2–4		Y	
3	5–7		Y	
2	8–10		Y	
1	>10		Y	
*Y: The parameters differentiate the control and CO_2_ stress treatments (*P* < 0.05).				
**CO_2_ specificity**	**Differentiate water stress treatment from**			
5	CO_2_ (≥10%) stress and water + CO_2_ stress^*^			
4	Water + CO_2_ stress			
3	CO_2_ (≥10%) stress			
2	Control			
1	N**			
*The parameters differentiate water stress treatment from compared treatments (*P* < 0.05); **N: The parameter cannot detect water stress.				
**Field applicability**	**Nondestructive**	**Remotely detectable**	**Automatic monitor**	**Continuous monitor**
5	Y*	Y	Y	Y
4	Y	Y	Y	N**
3	Y	Y	N	N
2	Y	N	N	N
1	N	N	N	N
*Y: the parameter meets the criterion; **N: the parameter does not meet the criterion.				
**Cost**	**Average cost of device and time**			
5	Low*			
4	Low + medium**			
3	Medium***			
2	Medium + high****			
1	High*****			
*Low: low device (<$5,000) and low time (<3 min).				
**Low + medium: (1) low device and medium time (4–20 min) or (2) low time and medium device ($5,000–$30,000).				
***Medium: (1) medium device and medium time, (2) low device and high time, or (3) low time and high device.				
****Medium + high: (1) medium device and high time (>20 min) or (2) medium time and high device (>$30,000).				
*****High: high device and high time.				

The first criterion of early detection was identified by the timing of when the parameter differentiated control and elevated soil CO_2_ treatment (Table 3). The time intervals for scoring were defined based on the observations of the timing with which plants respond to elevated soil CO_2_ concentrations in previous gassing studies (Patil, 2012; West et al., 2015; Lake et al., 2016b; He et al., 2019b). As 10% soil CO_2_ was reported as a threshold level to negatively affect plants (Al-Traboulsi et al., 2012; Jones et al., 2015; West et al., 2015), the timing for the significant difference between CO_2_ (≥10%) and control was used for scoring. Therefore, the parameter with a score of 5 was defined as capability to detect CO_2_ leakage within 1 day. Subsequently, the parameters with scores of 4, 3, 2, and 1 can detect leakage within 2–4 days, 5–7 days, 8–10 days, and >10 days, respectively (Patil, 2012; West et al., 2015; Lake et al., 2016b; He et al., 2019b).

The second criterion of CO_2_ specificity was identified by the ability to detect the difference between water and soil CO_2_ stresses, or between water and water + CO_2_ stresses. The identification of CO_2_ specificity was based on previous studies that reported similar responses of plants to water and CO_2_ stresses (≥40%) in the early stage of treatments (Lake et al., 2016b; Kim et al., 2017). Hence, the parameter, that differentiates water and CO_2_ stresses, could be expected to be CO_2_-specific. The parameter with a score of 5, in this criterion (Table 3), was defined by its capability to discriminate water stress from both 40% CO_2_ and water + CO_2_ stress (Bellante et al., 2014). On the other hand, the parameter with a score of 4 can differentiate water and 40% CO_2_ stress. The one with a score of 3 can differentiate water stress from mild CO_2_ stress (10% CO_2_). The parameter with a score of 2 can detect CO_2_ (≥10%) and water stresses but cannot differentiate both, while that with a score of 1 can only detect CO_2_ stress.

The third criterion of field applicability was identified by examining whether the parameter meets the four sub-standards, which are nondestructive, remotely detectable, automatic, and continuous measurements (Table 3). The selection of sub-standards of field applicability for each parameter was based on the literature and product catalogs (He et al., 2019a, 2019b; Li et al., 2013; Male et al., 2010; https://www.walz.com; https://www.malvernpanalytical.com). The nondestructive sampling of plants allows sustainable monitoring of CO_2_ leakage from plants (Noble et al., 2012). The remotely detectable parameters allow coverage of a large area (Male et al., 2010). Automatic and continuous measurements are helpful for ease of data collection and long-term monitoring in CCS sites (Male et al., 2010). The parameter with a score of 5 can meet four sub-standards, while the parameters with scores of 4, 3, 2, and 1 meet three, two, one, and none of the sub-standards, respectively.

The fourth criterion, cost, was identified by the average cost of sensor devices and time spent taking the measurement and data process (Table 3). The cost of devices was based on literature and sensor catalogs (Kim et al., 2019; Noble et al., 2012; https://www.walz.com; https://www.malvernpanalytical.com; https://licor.co.za) and the time cost for measurements was also referenced from above catalogs. In order to compare cost, the price of the device was categorized into three levels: low (<$5000), medium ($5000–$30,000), and high (>$30,000), following the extensive review by Noble et al. (2012), and the catalogs by the leading producers of sensors (https://www.walz.com; https://www.malvernpanalytical.com). In a similar manner, time for sampling and data processing was categorized into three levels, that is, low (<3 min), medium (4–20 min), and high (>20 min) (Noble et al., 2012). The parameter with a score of 5 (marked as low) requires that both the cost of device and time are classified as low level (Table 3). The parameter with a score of 4 (marked as low + medium) should be at least one low cost in device or time and one in medium cost in device or time. This included two conditions: (i) low price and medium time; (ii) low time and medium price (Table 3). The parameter with a score of 3 (marked as medium) should be the average cost of the device price and time at the medium level. This included three conditions: (i) medium cost in both device and time; (ii) low device price and high cost in time; and (iii) high device price and low cost in time. The parameter with a score of 2 (marked as medium + high) should be two conditions: (i) medium price and high time; and (ii) medium time and high price. The parameter with a score of 1 (marked as high) is high cost for both device and time (Table 3).

Finally, the IES value is calculated by adding the scores in the four criteria, following the equation:

(1) $${\rm IES} = \mathop \sum \limits_{{\rm i} = 1}^{\rm n} {\rm V}i$$

where V*i* is the score in the four criteria (*n* = 4) of early detection, CO_2_ specificity, field applicability, and cost.

### Statistical analysis

The light response curve of Y(II) to PAR for each leaf was processed in MATLAB R2014a (The MathWorks Inc., Natick, MA, USA). Analysis of variance (ANOVA) of Y(II), NDVI, PRI, EVI, NDWI, mNDWI, Pn, Gs, Tr, chlorophyl content, and biomass among the control, CO_2_, WATER, and WATER + CO_2_ treatments were analyzed using SAS 9.1 (SAS Institute Inc., Cary, NC, USA). The least-square means were used to test for significant differences among treatments at the 5% probability level. All results are reported as mean ± standard error.

## Results and discussions

### Soil conditions and morphological changes in plants

**Figure 2:**
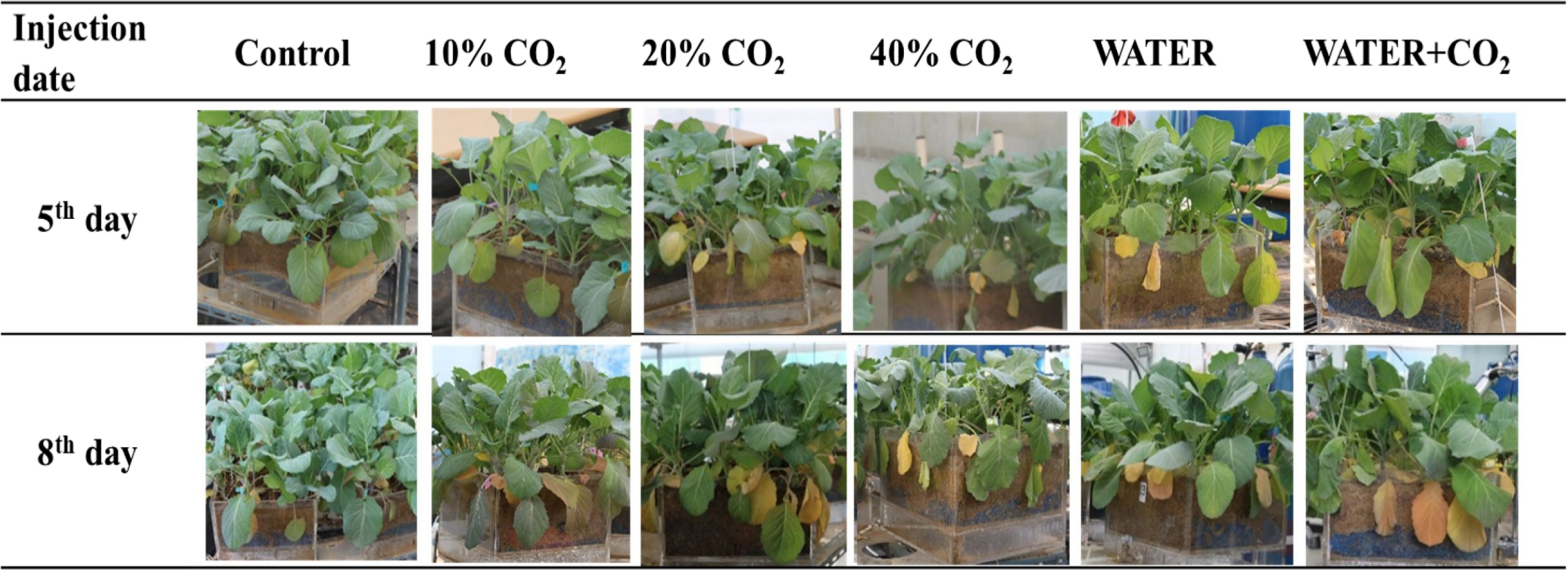
Plant visible changes in each treatment. The “injection date” in the 5th day and 8th day mean that the photos were taken at 5 and 8 days after CO_2_ injection started, respectively. The treatments included control, 10% CO_2_, 20% CO_2_, 40% CO_2_, water stress (WATER) and water stress combined CO_2_ stress (WATER + CO_2_).

In the 10%, 20%, and 40% CO_2_ treatments, soil gas concentrations were maintained at target levels during the experimental period (Table 4). Soil O_2_ concentrations decreased in all the CO_2_ treatments. This inference is consistent with the findings by Patil, Colls & Steven (2010), who found that the injected CO_2_ can replace soil O_2_. The photographs showed that the appearance of leaf chlorosis occurred in all the CO_2_ treatments on day 5, and yellow leaves significantly increased in the higher CO_2_ soil concentration treatments (Fig. 2). These results indicated that our experimental system was adequate for examining the effects of soil with different CO_2_ concentrations on plants.

**Table 4:** Soil CO_2_ concentration, O_2_ concentrations, and water contents.

Treatments	Soil CO_2_ (%)	Soil O_2_ (%)	Water content (WHC%)
Control	<1.0	21.4 (±0.02)	54.6 (±3.8)
10% CO_2_	10.2 (±0.27)	20.1 (±0.05)	54.8 (±3.7)
20% CO_2_	19.9 (±0.28)	19.0 (±0.07)	55.1 (±4.8)
40% CO_2_	40.0 (±0.29)	14.4 (±0.8)	56.0 (±5.4)
WATER	<1.0	21.5 (±0.01)	31.8 (±2.9)
WATER + CO_2_	39.9 (±0.25)	14.4 (±0.11)	31.9 (±4.3)

In the WATER and WATER + CO_2_ treatments, soil water content was maintained at the target levels during the experimental period (Table 4). The leaves in both treatments slightly wilted and drooped (Fig. 2). This indicated that those plants were subjected to water stress (Naser et al., 2010; Xavier et al., 2017). The photographs showed that water stress also induced leaf chlorosis (Fig. 2). The number of yellow leaves in the WATER + CO_2_ treatment was greater than those in the WATER treatment (Fig. 2), which implies that the effects of the combination of water and CO_2_ stresses on plants were worse than those of water stress.

### Parameters for early detection of CO_2_ leakage

**Figure 3:**
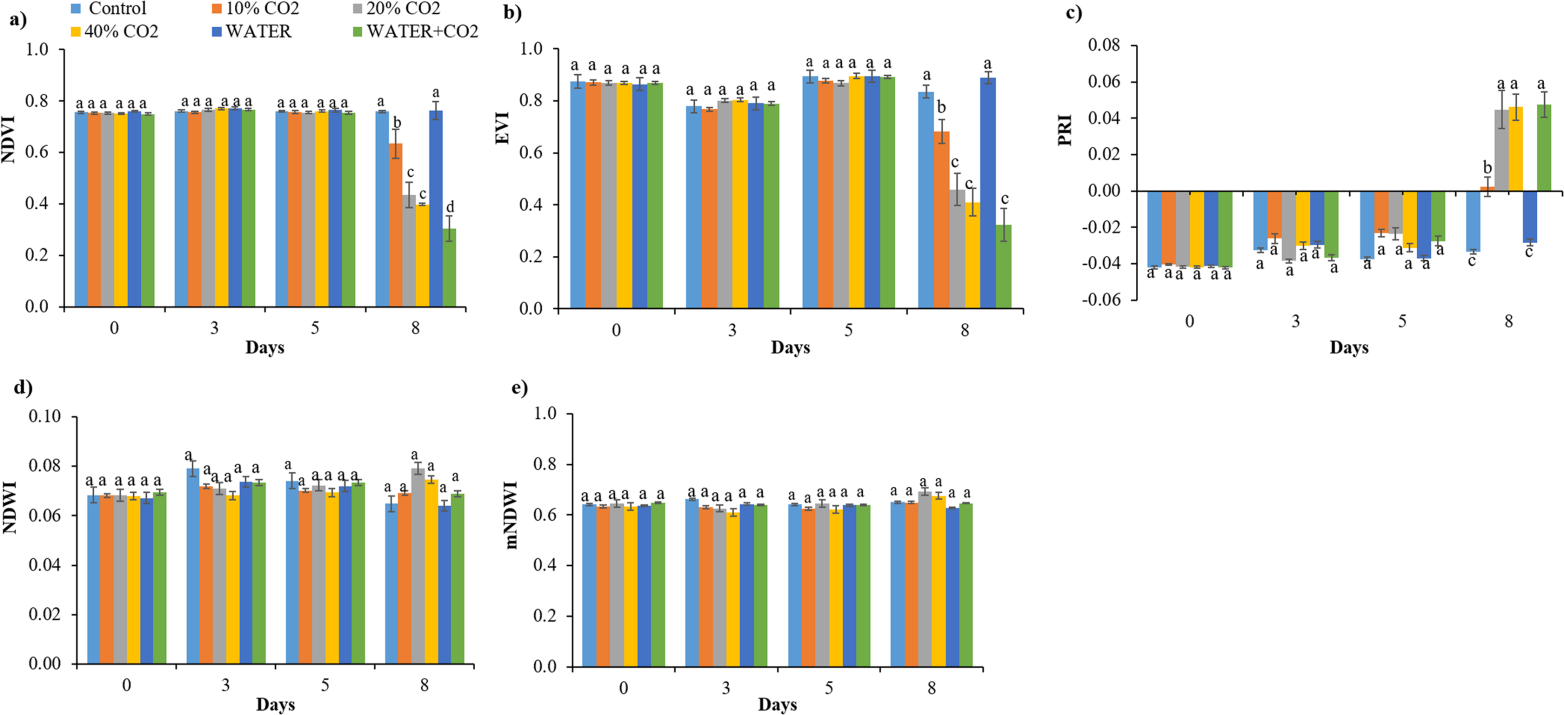
Values of vegetation indices of NDVI (A), EVI (B), PRI (C), NDWI (D), and mNDWI (E) in each treatment. Vertical lines represent the standard error (*n* = 30), and values for the same date with the same letter are not significantly different at a 5% significance level.

**Figure 4:**
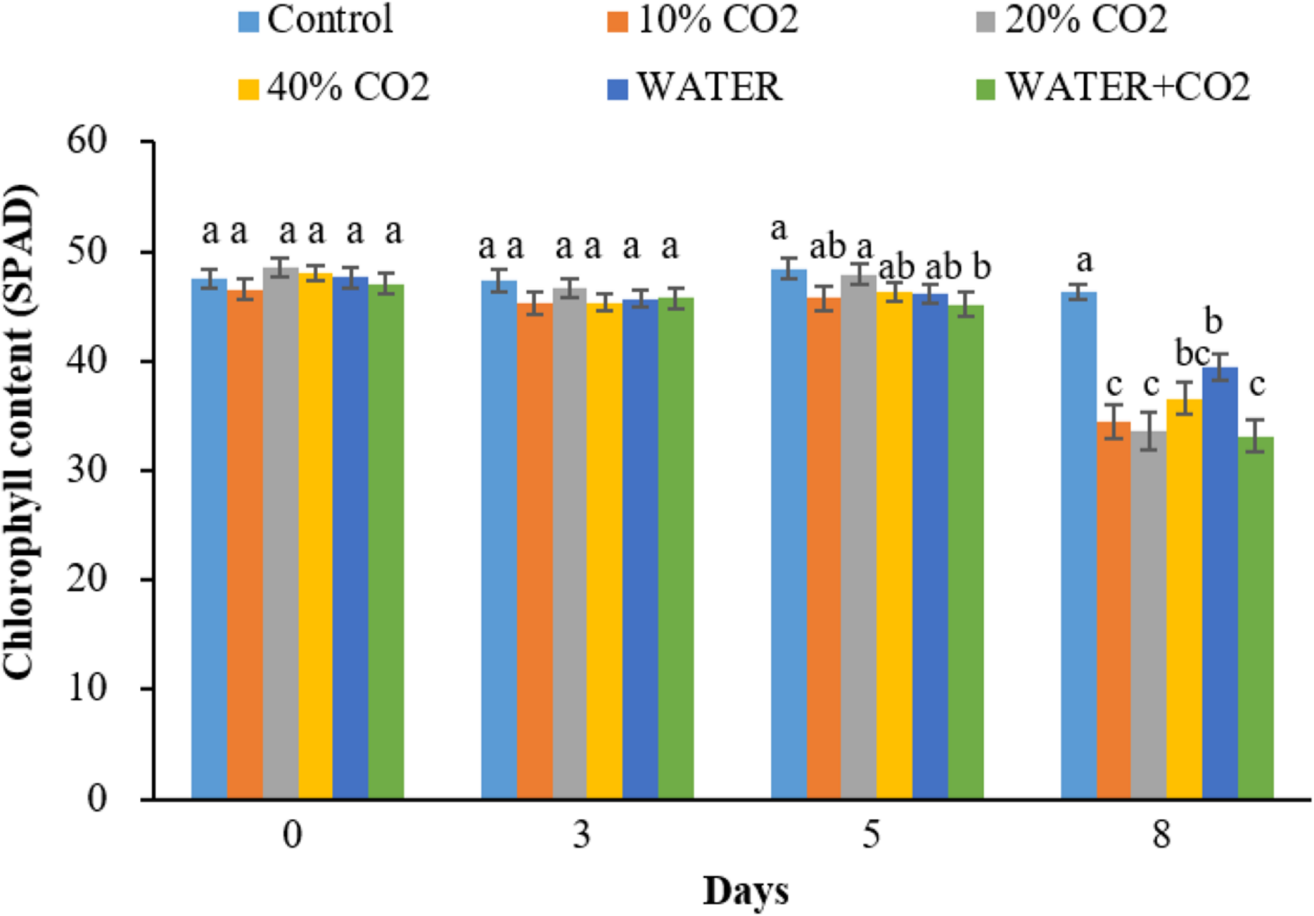
Chlorophyll contents change in each treatment over time. Vertical lines represent the standard error (*n* = 10), and values for the same date with the same letter are not significantly different at a 5% significance level.

The timing for the first differentiation between the control and CO_2_ treatments varied for different parameters. The hyperspectral reflectance parameters of NDVI, EVI, and PRI (within the pigment absorption bands) changed on day 8 in all CO_2_ treatments compared to the control (Fig. 3), while chlorophyl content first changed on day 5 in the 40% CO_2_ treatments (Fig. 4). These results imply that NDVI, EVI, PRI, and chlorophyl content can be used to monitor ecosystem changes by CO_2_ leakage, but their responses were not quick enough to detect CO_2_ leakage. Although the reflectance and chlorophyl parameters have been widely used to monitor CO_2_ leakage points in artificial gassing sites and natural CO_2_ spring areas (Bateson et al., 2008; Male et al., 2010; Patil, 2012; Feitz et al., 2014), the primary purpose of these parameters was not to detect CO_2_ leakage but to monitor overall changes in plants due to leakage.

**Figure 5:**
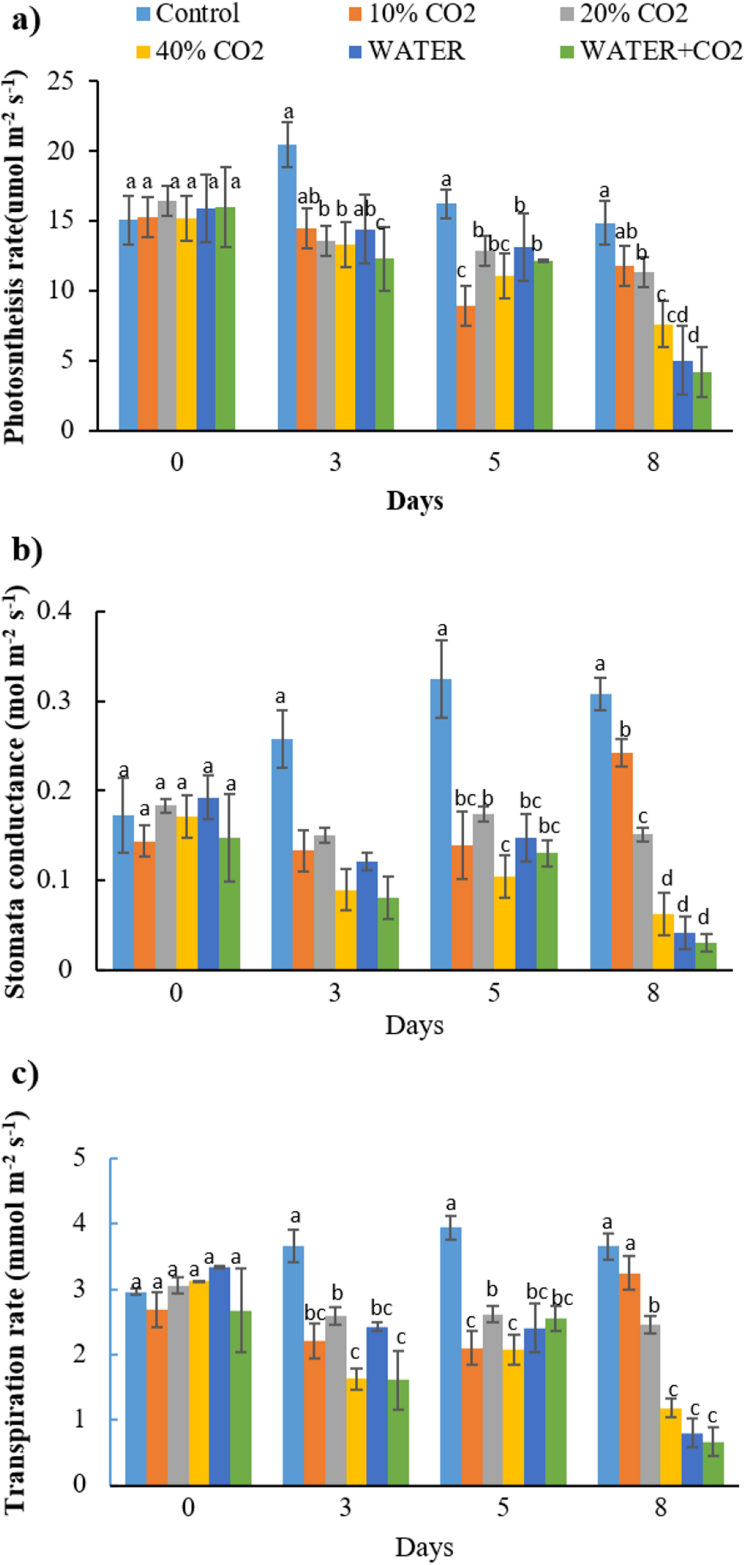
The changes of photosynthesis rate (A), stomata conductance (B), and transpiration rate (C) in each treatment (PAR = 1,000 μmol m^−2^ s^−1^). The ambient CO2 concentration (400 ppm) was used during the measurement. Vertical lines represent the standard error (*n* = 3) and values for the same date with the same letter are not significantly different at a 5% significance level.

As hypothesized, the responses of photosynthetic parameters (Pn, Gs, and Tr) to the elevated soil CO_2_ concentration were earlier than the changes in reflectance parameters and chlorophyl content. The changes in all the CO_2_ treatments were first observed on day 3 compared to those in the control, and the significant difference continued until the end of injection (Fig. 5; Table 5). The recovery of Pn, Gs, and Tr in 10% CO_2_ treatment from day 5 might be related to the plant compensation effects to the stress (Lake et al., 2016b; He et al., 2019a, 2019b). However, the overall lower values in the CO_2_ treatments than control indicated that CO_2_ leakage had adverse effects on the overall photosynthetic processes, which was consistent with the observations of Zhang et al. (2016). They reported that Pn, Gs, and Tr decreased after 4 days of CO_2_ leakage. According to Lake et al. (2016b), elevated soil CO_2_ concentration inhibits root water uptake and triggers the excretion of abscisic acid to close stomata, which instantly affects leaf photosynthesis and transpiration. Similar effects of elevated soil CO_2_ on roots and leaves were also reported by He et al. (2019b).

**Table 5:** Analysis of variance which examined the effects of CO_2_ stresses, water stress and water + CO_2_ stresses on plant parameters.

Source	Treatment	Date	Date × treatment
NDVI	<0.0001	<0.0001	<0.0001
EVI	<0.0001	<0.0001	<0.0001
PRI	<0.0001	<0.0001	<0.0001
NDWI	0.3882	0.0009	0.0002
mNDWI	0.2987	0.0007	0.0003
Chlorophyll content	<0.0001	<0.0001	<0.0001
Pn	0.0001	<0.0001	0.0401
Gs	<0.0001	0.1108	0.0002
Tr	<0.0001	0.0003	0.0001
Y(II)	<0.0001	<0.0001	<0.0001
Biomass	0.2726	–	–

**Figure 6:**
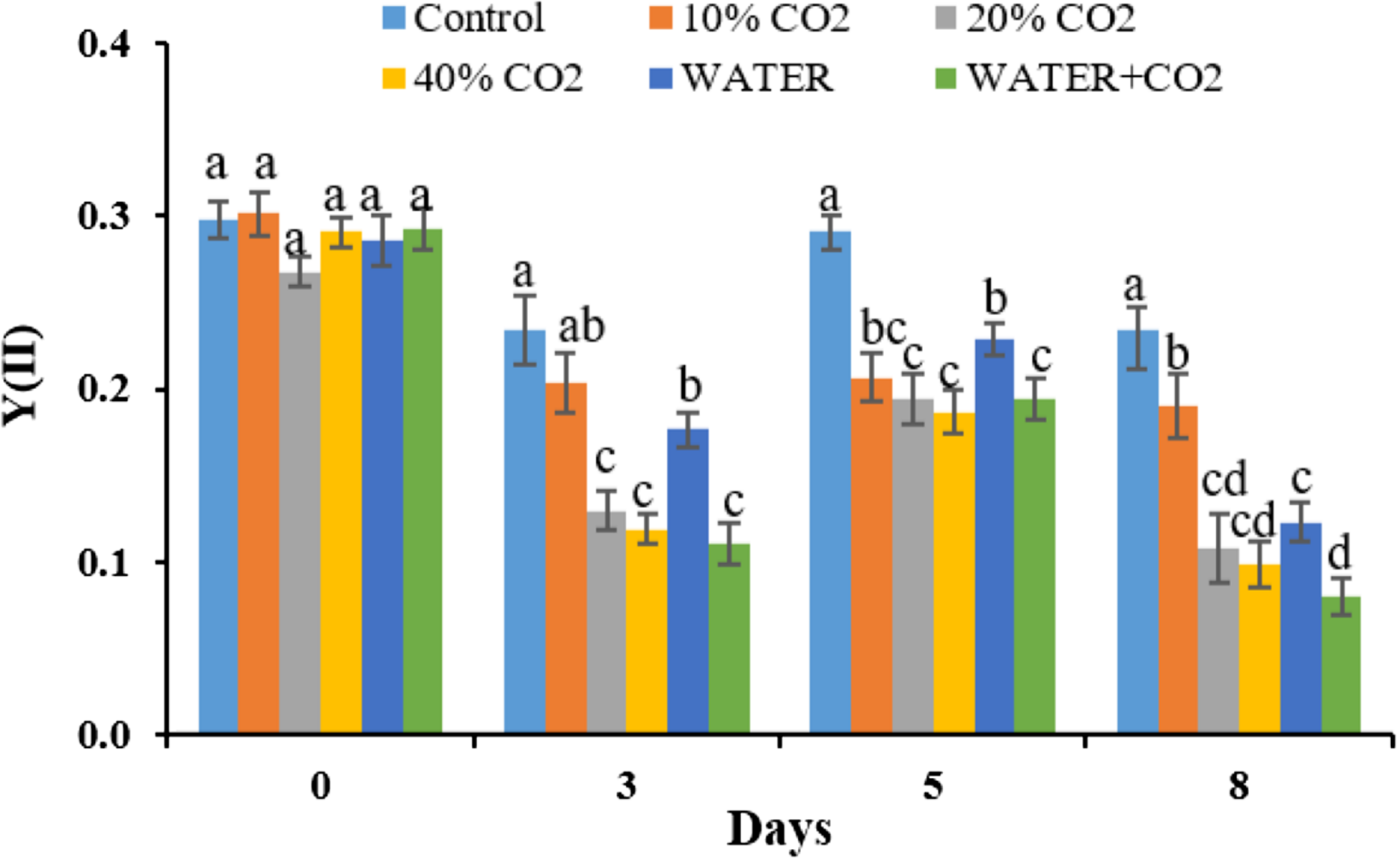
The changes of fluorescence parameters of Y(II) in each treatment (PAR = 1,000 μmol m^−2^ s^−1^). Vertical lines represent the standard error (*n* = 6), and values for the same date with the same letter are not significantly different at a 5% significance level.

Consistent with the photosynthetic process parameters, fluorescence parameters also showed early changes during the CO_2_ treatments (Figs. 5 and 6). On day 3, Y(II) in the 20% and 40% CO_2_ treatments were significantly lower than those in the control (Fig. 6). We consider Y(II) to be more sensitive than Pn, Gs, and Tr in detecting different levels of CO_2_ leakage because Y(II) differentiated 10% and 20% CO_2_ treatments on day 3, whereas Pn, Gs, and Tr did not.

**Figure 7:**
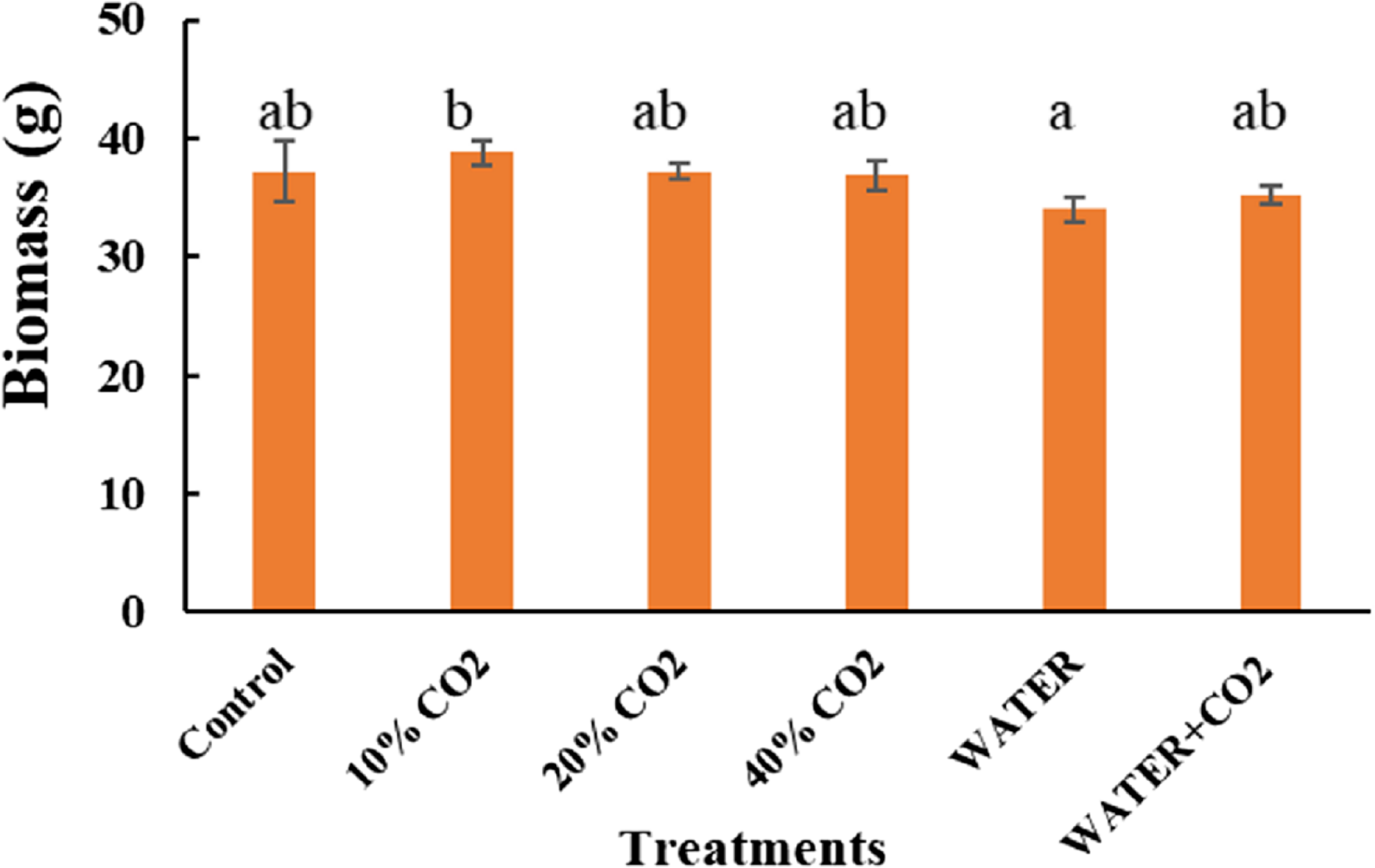
Plant biomass after injection stopped. Vertical lines represent the standard error (*n* = 3), and values for the same date with the same letter are not significantly different at a 5% significance level.

As expected, biomass did not change between the CO_2_ treatments and the control during this short-term incubation period. This indicates that the biomass is not helpful for early leakage detection and monitoring (Fig. 7). Consistent with our results, Kim et al. (2017) also reported no change in biomass after 10 days of 70% soil CO_2_ exposure.

Synthesizing the timing of the changes in all the parameters by CO_2_ treatments, the earliest responding parameters were Pn, Gs, Tr, and Y(II) (Figs. 5 and 6). Compared to reflectance parameters (NDVI, EVI, and PRI) and chlorophyl content, which initially differentiated control and 40% CO_2_ treatment on days 5 and 8 (Figs. 3 and 4), respectively, the Pn, Gs, Tr, and Y(II) were 2–5 days earlier. Our results are consistent with those of Lake et al. (2016b) and Zhang et al. (2016), who reported that the effects of elevated soil CO_2_ concentration on Pn, Gs, and Tr appeared within 4 days, which was earlier than that in chlorophyl content, observed within 7–15 days (Lakkaraju et al., 2010; Smith et al., 2017; He et al., 2019a, 2019b). As our experiment was the first to test the possibility of using fluorescence parameters to detect CO_2_ leakage, there is no reference with which to compare our results. However, Rossini et al. (2015) reported that the effect of herbicide on chlorophyl fluorescence parameters was earlier than that on NDVI. In summary, we suggest that Pn, Gs, Tr, and Y(II) are early indicators of CO_2_ leakage monitoring.

### CO_2_ specific parameters for differentiation of CO_2_ and water stresses

In the WATER treatment, the parameters of chlorophyl content, Pn, Gs, Tr, and Y(II) were significantly lower than those in the control from day 3 to day 8 (Figs. 4–6; Table 5), indicating that these parameters can be used as indicators for detecting water stress. These results of chlorophyl content, Pn, Gs, and Tr (Figs. 4 and 5) are consistent with previous observations that water stress would lead to leaf stomata closure and restrict CO_2_ diffusion into the chloroplast to affect photosynthesis (Kozlowsk, 1972). The Y(II) were also reported that can differentiate plants in moderate or excessive drought stresses from those in non-stressed conditions (Li et al., 2013; Guo & Tan, 2015; Xavier et al., 2017). Unexpectedly, leaf-level measured NDWI and mNDWI (within the water-absorption bands) failed to detect water stress (Fig. 3). This result is inconsistent with the findings of Male et al. (2010) that canopy-level measured NDWI observed leaf water content reduction in a pasture field after the long, hot summer. Compared to Male et al. (2010), we treated broadleaf plants with mild water stress over a short period (10 days). Ling et al. (2019) reported that the leaf-level spectral measurement may be affected by the stress status and plant species due to different leaf structures, age, and front or back sides of leaves. Hence, we argue that NDWI and mNDWI may not detect short-term mild water stress in plants.

The negative effects of water stress on plant chlorophyl content, Pn, Gs, Tr, and Y(II) could be similar to those of CO_2_ stress, at least in the initial stage, because the mechanism how underground CO_2_ influence plant is related to plants’ root water absorption. He et al. (2019b) reported that high soil CO_2_ could reduce root water absorption activity inducing leaf chlorophyl reduction. Lake et al. (2016b) explained the mechanism of how high soil CO_2_ influence plant photosynthesis. The similarity between water and CO_2_ stresses in early stage of exposure makes CO_2_ leakage monitoring using plant more difficult in the field. The Y(II), differentiated WATER from 40% CO_2_, and WATER from WATER+ CO_2_ (Fig. 6). This verifies that chlorophyl fluorescence would be very helpful in distinguishing plants living in the CO_2_ leakage area, which also suffered from water stress. Although chlorophyl content did not differentiate WATER from 40% CO_2_ and WATER from WATER + CO_2_, it differentiated 40% CO_2_ from WATER treatments. This still implies that this would be useful in distinguishing water stress from extreme CO_2_ stress in the field. On the other hand, the parameters of Pn, Gs, Tr had significant differences between any CO_2_ treatment (≥10%) and WATER treatment (Fig. 5), which indicates that they could differentiate the effects of mild CO_2_ leakage from water stress.

### Field applicability and cost of the parameters

The field applicability of each parameter varied (Table 6). The fluorescence parameters of Y(II) and reflectance parameters of NDVI, EVI, PRI NDWI, and mNDWI had high field applicability because they can automatically and continuously monitor plant conditions without destruction (Delegido et al., 2011; Gautam et al., 2015; Janka et al., 2015) (Table 6). In particular, the reflectance parameters were remotely measurable in the artificial gassing sites by installing sensors on unmanned aerial vehicles (Male et al., 2010; Feitz et al., 2014). The photosynthetic parameters of Pn, Gs, and Tr and transmittance parameters of chlorophyll content had limited field applicability because these parameters were only measured at the leaf level, although the measurements would not destroy plants (Wu et al., 2014; Kim et al., 2017). Finally, biomass measurement destroyed plants and could not be remotely, automatically, and continuously detected, which had the lowest field applicability (He et al., 2016) (Table 6).

**Table 6:** Field applicability of the parameters.

Parameters	Measurement principles	Measurement devices	Nondestructive	Remotely detectable	Automatic monitor	Continuous monitor	References
NDVI	Reflectance	Spectrometer	Y	Y	Y	Y	Cheng et al. (2009), Delegido et al. (2011), Gautam et al. (2015), Kim et al. (2019), Male et al. (2010), Penuelas et al. (1997), Ryu et al. (2014); https://handheld.psi.cz; https://www.oceaninsight.com; http://en.li-ca.com
EVI							
PRI							
NDWI							
mNDWI							
Chlorophyll content	Transmittance	Photodiode detector	Y	N	N	N	Süß et al. (2015), https://www.specmeters.com, https://www.konicaminolta.com
Pn	Absorption of infrared radiation	Infrared gas analyzer	Y	N	N	N	Paul et al. (2017), Spangler et al. (2009), https://licor.co.za
Gs							
Tr							
Y(II)	Fluorescence emission	Fluorometer	Y	N	Y	Y	Janka et al. (2015), Porcar-Castell et al. (2008), Rossini et al. (2015), https://www.walz.com; https://fluorometers.psi.cz; https://www.hansatech-instruments.com
Biomass	Biomass	Oven and scale	N	N	N	N	He et al. (2016), https://www.thermofisher.com

Although the range of the device prices is large and there could be cheap sensors that are not commercially manufactured, we took the median price for simplicity (the median value between the lowest and highest prices) to evaluate and compare the cost of sensors. Based on our evaluation standards (Table 3), the parameters with low device cost were chlorophyl content and biomass because their median device prices were <$5,000 (Table 7) (https://www.specmeters.com; https://www.konicaminolta.com). The parameters of NDVI, EVI, PRI, NDWI, mNDWI, and Y(II) had medium device cost because the median price of the spectrometer and fluorometer was approximately $26,000 (i.e., ranged from $1,000 to $50,000) and $23,000 (i.e., ranged from $6,000 to $40,000), respectively (Table 7) (https://www.walz.com; https://fluorometers.psi.cz; https://www.hansatech-instruments.com; https://handheld.psi.cz; https://www.oceaninsight.com; http://en.li-ca.com). Finally, the parameters with high device cost were Pn, Gs, and Tr because the price LI-6400 or LI-6800 generally ranged between $40,000 and $60,000 (Table 7) (https://licor.co.za).

**Table 7:** Device and time costs of parameters.

Parameters	Devices	Median device cost ($)	Time cost for sampling and data process (min/sample)	Average cost of device and time
NDVI	Spectrometer	Medium*	Medium**	Medium***
EVI				
PRI				
NDWI				
mNDWI				
Chlorophyll content	Chlorophyll meter	Low	Low	Low
Pn	LI-6400/6800	High	High	High
Gs				
Tr				
Y(II)	Fluorometer	Medium	Medium	Medium
Biomass	Oven	Low	High	Medium

**Notes:**

* Device cost: low (<$5,000), medium ($5,000–$30,000), and high (>$30,000).

** Time cost: low (<3 min), medium (4–20 min), and high (>20 min).

*** Average cost: based on the evaluation standards in Table 3.

The parameter with low time cost was chlorophyl content because the total time for sampling and data processing ranged from a few seconds to 1 min (Table 7). The parameters with medium time cost were NDVI, EVI, PRI, NDWI, mNDWI, and Y(II). Their sampling times ranged from 10 s to 5 min (depending on sensor setup and user skills), and those of data processing time ranged from 3 min to 10 min (including MATLAB code writing) (Table 7). Finally, the parameters of Pn, Gs, and Tr and biomass had a high time cost due to the long sampling time (>20 min) (Table 7). After averaging the cost of device and time for each parameter, we can suggest chlorophyl content as a low-cost parameter for leakage monitoring (Table 7).

### Indicator evaluation score of parameters and their applications

As our four criteria have equal importance, we did not give different weightings on them and we treated four criteria equal in mathematical way. So, the highest score means the best parameter. However, we did not only focus on the best score because we realize that the second or third best choices could also be useful in certain circumstances. Hence, we keep the scores of four criteria in the Table 8 and let users decide which would be the best choice of theirs depending on the conditions.

**Table 8:** Scores and IES values of parameters.

Parameters	Early detection	CO_2_ specificity	Field applicability	Cost	IES
Y(II)	4	5	4	3	16
NDVI	2	5	5	3	15
EVI	2	5	5	3	15
PRI	2	5	5	3	15
NDWI	1	1	5	3	10
mNDWI	1	1	5	3	10
Pn	4	2	2	1	9
Gs	4	2	2	1	9
Tr	4	2	2	1	9
Chlorophyll content	3	4	2	5	14
Biomass	1	1	1	3	6

The fluorescence parameters of Y(II) had the highest IES values (Table 8), which suggests that it is the most efficient parameters. They had high scores in the criteria of early detection, CO_2_ specificity, and field applicability, although the cost of measurement is considered to be medium (Table 8). Murchie & Lawson (2013) also suggested Y (II) as a useful indicator for field monitoring because it shows a rapid response to environmental stresses. Furthermore, the measurement of Y(II) does not require waiting for dark adaptation of plants, and thus has a fast detection. The second-highest IES values were the reflectance parameters of NDVI, EVI, and PRI (Table 8), which were still useful for CO_2_ leakage monitoring. NDVI, EVI, and PRI had the highest score in field applicability and have been successfully applied in field detection (Male et al., 2010; Wimmer et al., 2011; Feitz et al., 2014). The third-highest IES was the SPAD-measured chlorophyl content, which has the major advantage of low cost; however, it has limited applicability to the laboratory or greenhouse (Table 8). The photosynthetic parameters of Pn, Gs, and Tr and reflectance parameters of NDWI and mNDWI had relatively low IES values (Table 8); therefore, they were less efficient for CO_2_ leakage monitoring. Although Pn, Gs, and Tr were able to detect CO_2_ leakage early, they did not differentiate CO_2_ stress from other stresses. Moreover, the measurement can only be done at the leaf level, and the time and labor of measurement are not cost-effective. Compared to the photosynthetic parameters, NDWI and mNDWI can be applied to the field scale because they can cover large area of canopy by loading the sensor on the unmanned vehicles or drone. However, their ability to detect early leakage and accurately differentiate CO_2_ stress was minimal. Finally, biomass had the lowest IES value, which indicates that it is not suitable for CO_2_ leakage monitoring because it had the lowest scores in all the criteria except cost (Table 8).

## Conclusion

In this study, we tested the possibility of using fluorescence parameters as a proper plant indicator to detect CO_2_ leakage. To the best of our knowledge, this is the first study to suggest chlorophyl fluorescence parameter as a useful plant indicator in CCS sites. This parameter was made convenient by the advanced fluorometer of MONI-PAM, which can detect fluorescence without dark adaptation. The Y(II) detected the treatment effect of soil at 10–40% CO_2_ concentrations early and differentiated CO_2_ and water stresses, establishing them as early and CO_2_ specific parameters for leakage monitoring.

The IES method developed to compare the ability and applicability of plant parameters for CO_2_ leakage monitoring, was sufficiently systematic to be used as a guideline for environmental management in CCS sites. Following the IES results, we suggest that the chlorophyl fluorescence parameters of Y(II) is the most efficient indicators for detecting CO_2_ leakage in the field. Although the reflectance parameters of NDVI, EVI, and PRI did not detect CO_2_ leakage early, they are still useful for the large-area monitoring of CO_2_ leakage points in CCS sites. The photosynthetic parameters and chlorophyl content were found to be unsuitable for field monitoring, but useful to measure the early response of plants to CO_2_ leakage in small-scale studies.

## Supplemental Information

10.7717/peerj.10652/supp-1 Supplemental Information 1 The data of leaf chlorophyl content, photosynthesis and fluorescence of each treatment measured during experimental period. Give data show raw value of leaf chlorophyl content, photosynthesis and fluorescence measured by sensors. The treatments included control, 10% CO_2_ (10% soil CO_2_), 20% CO_2_ (20% soil CO_2_), 40% CO_2_ (40% soil CO_2_), water stress (WATER) and water stress with 40% CO_2_ treatment (WATER + CO_2_). The measuring date was October 10, 13, 15, and 18 (i.e., 0, 3, 5, and 8 days after experiment onset, respectively), 2018.

10.7717/peerj.10652/supp-2 Supplemental Information 2 The leaf spectral reflectance data of each treatment measured during experimental period. Give data show raw value of leaf spectral reflectance values measured by sensors. The treatments included control, 10% CO_2_ (10% soil CO_2_), 20% CO_2_ (20% soil CO_2_), 40% CO_2_ (40% soil CO_2_), water stress (WATER) and water stress with 40% CO_2_ treatment (WATER + CO_2_). The measuring date was October 10, 13, 15, and 18 (i.e., 0, 3, 5, and 8 days after experiment onset, respectively), 2018.

## Additional Information and Declarations

### Data Availability

The following information was supplied regarding data availability: Raw data, including record of the changes of plant parameter after stress treatment, are available in the Supplemental Files.

## References

[ref-1] Al-Traboulsi M, Sjögersten S, Colls J, Steven M, Craigon J, Black C (2012). Potential impact of CO_2_ leakage from carbon capture and storage (CCS) systems on growth and yield in spring field bean. Environmental and Experimental Botany.

[ref-2] Baker NR (2008). Chlorophyll fluorescence: a probe of photosynthesis in vivo. Annual Review of Plant Biology.

[ref-3] Bateson L, Vellico M, Beaubien SE, Pearce JM, Annunziatellis A, Ciotoli G, Coren F, Lombardi S, Marsh S (2008). The application of remote-sensing techniques to monitor CO2-storage sites for surface leakage: method development and testing at Latera (Italy) where naturally produced CO2 is leaking to the atmosphere. International Journal of Greenhouse Gas Control.

[ref-4] Beaubien SE, Ciotoli G, Coombs P, Dictor MC, Krüger M, Lombardi S, Pearce JM, West JM (2008). The impact of a naturally occurring CO_2_ gas vent on the shallow ecosystem and soil chemistry of a Mediterranean pasture (Latera, Italy). International Journal of Greenhouse Gas Control.

[ref-5] Bellante GJ, Powell SL, Lawrence RL, Repasky KS, Dougher T (2014). Hyperspectral detection of a subsurface CO2 leak in the presence of water stressed vegetation. PLOS ONE.

[ref-6] Cartier K (2020). Basalts turn carbon into stone for permanent storage. *Eos*.

[ref-7] Casanova-katny A, Barták M, Gutierrez C (2019). Open top chamber microclimate may limit photosynthetic processes in Antarctic lichen: case study from King George. Czech Polar Reports.

[ref-8] Chen F, Zhang W, Ma J, Yang Y, Zhang S, Chen R (2017). Experimental study on the effects of underground CO_2_ leakage on soil microbial consortia. International Journal of Greenhouse Gas Control.

[ref-71] Cheng Y-B, Middleton EM, Hilker T, Coops NC, Black TA, Krishnan P (2009). Dynamics of spectral bio-indicators and their correlations with light use efficiency using directional observations at a douglas-fir forest. Measurement Science and Technology.

[ref-9] Delegido J, Verrelst J, Alonso L, Moreno J (2011). Evaluation of sentinel-2 red-edge bands for empirical estimation of green LAI and chlorophyll content. Sensors.

[ref-10] European Commission & European Communities (2011). Implementation of directive 2009/31/EC on the geological storage of carbon dioxide—guidance document 2—characterisation of the storage complex, CO2 stream composition, monitoring and corrective measures.

[ref-11] Feitz A, Jenkins C, Schacht U, McGrath A, Berko H, Schroder I, Noble R, Kuske T, George S, Heath C, Zegelin S, Curnow S, Zhang H, Sirault X, Jimenez-Berni J, Hortle A, Henry B, Schroder I, Noble R, Kuske T, George S, Charles H, Zegelin S, Cumow S, Zhang H, Sirault X, Jimenez-Berni J, Hortle A (2014). An assessment of near surface CO2 leakage detection techniques under Australian conditions. Energy Procedia.

[ref-12] Gamon JA, Peñuelas J, Field CB (1992). A narrow-waveband spectral index that tracks diurnal changes in photosynthetic efficiency. Remote Sensing of Environment.

[ref-69] Gao BC (1996). NDWI—A normalized difference water index for remote sensing of vegetation liquid water from space. Remote Sensing of Environment.

[ref-13] Gautam VK, Gaurav PK, Murugan P, Annadurai M (2015). Assessment of surface water dynamicsin Bangalore using WRI, NDWI, MNDWI, supervised classification and K-T transformation. Aquatic Procedia.

[ref-14] Guo Y, Tan J (2015). Recent advances in the application of chlorophyll a fluorescence from photosystem II. Photochemistry and Photobiology.

[ref-15] Hatfield JL, Gitelson AA, Schepers JS, Walthall CL (2008). Application of spectral remote sensingfor agronomic decisions. Agronomy Journal.

[ref-16] He W, Kim Y, Ko D, Yun S, Jun S, Yoo G (2019a). Changes in soil N2O and CH4 emissions and related microbial functional groups in an artificial CO2 gassing experiment. Science of the Total Environment.

[ref-17] He W, Moonis M, Chung H, Yoo G (2016). Effects of high soil CO_2_ concentrations on seed germination and soil microbial activities. International Journal of Greenhouse Gas Control.

[ref-18] He W, Yoo G, Moonis M, Kim Y, Chen X (2019b). Impact assessment of high soil CO_2_ on plant growth and soil environment: a greenhouse study. PeerJ.

[ref-68] Huete A, Didan K, Miura T, Rodriguez EP, Gao X, Ferreira LG (2002). Overview of the radiometric and biophysical performance of the MODIS vegetation indices. Remote Sensing of Environment.

[ref-19] IPCC (2005). Intergovernmental panel on climate change, carbon dioxide capture and storage.

[ref-20] Pachauri RK, Meyer LA (2014). IPCC, 2014: Climate Change 2014: Mitigation of Climate Change. Contribution of Working Group III to the Fifth Assessment Report of the Intergovernmental Panel on Climate Change.

[ref-21] Janka E, Körner O, Rosenqvist E, Ottosen C-O (2015). Using the quantum yields of photosystem II and the rate of net photosynthesis to monitor high irradiance and temperature stress in chrysanthemum (Dendranthema grandiflora). Plant Physiology and Biochemistry.

[ref-22] Jiang Q, Roche D, Monaco TA, Durham S (2006). Gas exchange, chlorophyll fluorescence parameters and carbon isotope discrimination of 14 barley genetic lines in response to salinity. Field Crops Research.

[ref-23] Jiang J, Steven MD, He R, Chen Y, Du P, Guo H (2015). Identifying the spectral responses of several plant species under CO_2_ leakage and waterlogging stresses. International Journal of Greenhouse Gas Control.

[ref-24] Jones DG, Barkwith AKAP, Hannis S, Lister TR, Gal F, Graziani S, Beaubien SE, Widory D (2014). Monitoring of near surface gas seepage from a shallow injection experiment at the CO2 Field Lab, Norway. International Journal of Greenhouse Gas Control.

[ref-25] Jones DG, Beaubien SE, Blackford JC, Foekema EM, Lions J, De Vittor C, West JM, Widdicombe S, Hauton C, Queirós AM (2015). Developments since 2005 in understanding potential environmental impacts of CO2 leakage from geological storage. International Journal of Greenhouse Gas Control.

[ref-26] Kalaji HM, Račková L, Paganová V, Swoczyna T, Rusinowski S, Sitko K (2018). Can chlorophyll-a fluorescence parameters be used as bio-indicators to distinguish between drought and salinity stress in Tilia cordata Mill?. Environmental and Experimental Botany.

[ref-27] Kim Y, He W, Ko D, Chung H, Yoo G (2017). Increased N2O emission by inhibited plant growth in the CO2 leaked soil environment: Simulation of CO2 leakage from carbon capture and storage (CCS) site. Science of the Total Environment.

[ref-28] Kim J, Ryu Y, Jiang C, Hwang Y (2019). Continuous observation of vegetation canopy dynamics using an integrated low-cost, near-surface remote sensing system. Agricultural and Forest Meteorology.

[ref-29] Kozlowsk TT (1972). Water deficits and plant growth.

[ref-30] Krüger M, Jones D, Frerichs J, Oppermann BI, West J, Coombs P, Green K, Barlow T, Lister R, Shaw R, Strutt M, Möller I (2011). Effects of elevated CO_2_ concentrations on the vegetation and microbial populations at a terrestrial CO_2_ vent at Laacher See, Germany. International Journal of Greenhouse Gas Control.

[ref-31] Lake J, Smith K, Steven M, Lomax B (2013). COOLTRANS—environmental impacts of CO2 leakage into the soil environment.

[ref-32] Lake J, Steven M, Smith K, Lomax B (2016a). Plant responses to elevated CO_2_ levels in soils: distinct CO_2_ and O_2_-depletion effects. International Journal of Greenhouse Gas Control.

[ref-33] Lake J, Walker HJ, Cameron DD, Lomax BH (2016b). A novel root-to-shoot stomatal response to very high CO_2_ levels in the soil: electrical, hydraulic and biochemical signalling. Physiologia Plantarum.

[ref-34] Lakkaraju VR, Zhou X, Apple ME, Cunningham A, Dobeck LM, Gullickson K, Spangler LH (2010). Studying the vegetation response to simulated leakage of sequestered CO_2_ using spectral vegetation indices. Ecological Informatics.

[ref-35] Li GL, Wu HX, Sun YQ, Zhang SY (2013). Response of chlorophyll fluorescence parameters to drought stress in sugar beet seedlings. Russian Journal of Plant Physiology.

[ref-36] Ling B, Goodin DG, Raynor EJ, Joern A (2019). Hyperspectral analysis of leaf pigments and nutritional elements in tallgrass prairie vegetation. Frontiers in Plant Science.

[ref-37] Male EJ, Pickles WL, Silver EA, Hoffmann GD, Lewicki J, Apple M, Repasky K, Burton EA (2010). Using hyperspectral plant signatures for CO_2_ leak detection during the 2008 ZERT CO_2_ sequestration field experiment in Bozeman, Montana. Environmental Earth Sciences.

[ref-38] Maxwell K, Johnson GN (2000). Chlorophyll fluorescence—a practical guide. Journal of Experimental Botany.

[ref-39] Mendelssohn IA, McKee KL, Kong T (2001). A comparison of physiological indicators of sublethal cadmium stress in wetland plants. Environmental and Experimental Botany.

[ref-40] Murchie EH, Lawson T (2013). Chlorophyll fluorescence analysis: a guide to good practice and understanding some new applications. Journal of Experimental Botany.

[ref-41] Narayan A, Misra M, Singh R, Misra AN (2012). Chlorophyll fluorescence in plant biology. Biophysics.

[ref-42] Naser L, Kourosh V, Bahman K, Reza A (2010). Soluble sugars and proline accumulation play a role as effective indices for drought tolerance screening in Persian walnut (Juglans regia L.) during germination. Fruits.

[ref-43] Noble RRP, Stalker L, Wakelin SA, Pejcic B, Leybourne MI, Hortle AL, Michael K (2012). Biological monitoring for carbon capture and storage: a review and potential future developments. International Journal of Greenhouse Gas Control.

[ref-44] Patil RH, Mahamane A (2012). Impacts of carbon dioxide gas leaks from geological storage sites on soil ecology and above ground vegetation. Diversity of Ecosystems.

[ref-45] Patil R, Colls J, Steven M (2010). Effects of CO_2_ gas as leaks from geological storage sites on agro-ecosystems. Energy.

[ref-74] Paul V, Pandey R, Sharma L, Meena RC (2017). Physiological techniques to analyze the impact of climate change on crop plants. Division of Plant Physiology.

[ref-46] Pearce J, Blackford J, Beaubien S, Foekema E, Gemeni V, Gwosdz S, Jones D, Kirk K, Lions J, Metcalfe R, Moni C, Smith K, Steven M, West J, Ziogou F, Blackford J, Beaubien S, Foekema E, Gemeni V, Kirk K, Lions J, Metcalfe R, Moni C, Smith K, Stevens M, West J, Ziogou F (2014). A guide for assessing the potential impacts on ecosystems of leakage from CO2 storage sites. Energy Procedia.

[ref-47] Penuelas J, Llusia J, Pinol J, Filella I (1997). Photochemical reflectance index and leaf photosynthetic radiation-use-efficiency assessment in Mediterranean trees. International Journal of Remote Sensing.

[ref-48] Pfanz H, Vodnik D, Wittmann C, Aschan G, Raschi A, Esser K, Lüttge U, Beyschlag W (2004). Plants and geothermal CO2 exhalations—survival in and adaptation to a high CO2 environment. Progress in Botany.

[ref-49] Porcar-Castell A, Pfündel E, Korhonen JFJ, Juurola E (2008). A new monitoring PAM fluorometer (MONI-PAM) to study the short- and long-term acclimation of photosystem II in field conditions. Photosynthesis Research.

[ref-50] Pruess K (2011). Integrated modeling of CO_2_ storage and leakage scenarios including transitions between super- and subcritical conditions, and phase change between liquid and gaseous CO_2_. Greenhouse Gases: Science and Technology.

[ref-51] Roháček K, Soukupová J, Barták M, Schoefs B (2008). Chlorophyll fluorescence: a wonderful tool to study plant physiology and plant stress. Plant Cell Compartments—Selected Topics.

[ref-70] Rossini M, Nedbal L, Guanter L, AAč Alonso, Burkart L, Cogliati A, Colombo S, Damm R, Drusch A, Hanus M, Janoutova J, Julitta R, Kokkalis T, Moreno P, Novotny J, Panigada J, Pinto C, Schickling F, Zemek F, Rascher U (2015). Red and far red Sun-induced chlorophyll fluorescence as a measure of plant photosynthesis. Geophysical Research Letters.

[ref-72] Ryu Y, Lee G, Jeon S, Song Y, Kimm H (2014). Monitoring multi-layer canopy spring phenology of temperate deciduous and evergreen forests using low-cost spectral sensors. Remote Sensing of Environment.

[ref-52] Sharma B, Apple ME, Zhou X, Olson JM, Dorshorst C, Dobeck LM, Cunningham AB, Spangler LH (2014). Physiological responses of dandelion and orchard grass leaves to experimentally released upwelling soil CO_2_. International Journal of Greenhouse Gas Control.

[ref-53] Smith KL, Lake JA, Steven MD, Lomax BH (2017). Effects of elevated soil CO2 concentration on growth and competition in a grass-clover mix. International Journal of Greenhouse Gas Control.

[ref-73] Spangler LH, Dobeck LM, Repasky KS, Nehrir AR, Humphries SD, Barr JLJL, Keith CJ, Shaw JA, Rouse JH, Cunningham AB, Benson SM, Oldenburg CM, Lewicki JL, Wells AW, Diehl JR, Strazisar BR, Fessenden JE, Rahn Ta, Amonette JE, Barr JL, Pickles WL, Jacobson JD, Silver EA, Male EJ, Rauch HW, Gullickson KS, Trautz R, Kharaka Y, Birkholzer J, Wielopolski L (2009). A shallow subsurface controlled release facility in, for testing near surface CO2 detection techniques and transport models. Environmental Earth Sciences.

[ref-54] Süß A, Danner M, Obster C, Locherer M, Hank T, Richter K (2015). Measuring leaf chlorophyll content with the Konica Minolta SPAD-502Plus EnMAP field guides technical report. https://gfzpublic.gfz-potsdam.de/rest/items/item_1388302/component/file_1388303/content.

[ref-55] Sven B, Mates B, John B (2014). Photosynthesis in the marine environment. Oceanography.

[ref-56] Tang Y, Gao F, Guo S, Li F (2014). Effects of hypobaria and hypoxia on seed germination of six plant species. Life Sciences in Space Research.

[ref-57] Tucker CJ (1979). Red and photographic infrared linear combinations for monitoring vegetation. Remote Sensing of Environment.

[ref-58] Vodnik D, Pfanz H, Wittmann C, Maček I, Kastelec D, Turk B, Batič F (2002). Photosynthetic acclimation in plants growing near a carbon dioxide spring. Phyton—Annales Rei Botanicae.

[ref-59] Vrålstad T, Todorovic J, Wollenweber J, Abdollahi J, Karas D, Buddensiek M (2018). MiReCOL report D8.1: description of leakage scenarios for consideration in the work in SP3. Trondheim, Norway. https://www.mirecol-co2.eu/download/D08.1%20-%20Description%20of%20leakage%20scenarios.pdf.

[ref-60] West JM, Jones DG, Annunziatellis A, Barlow TS, Beaubien SE, Bond A, Breward N, Coombs P, De Angelis D, Gardner A, Gemeni V, Graziani S, Green KA, Gregory S, Gwosdz S, Hannis S, Kirk K, Koukouzas N, Krüger M, Libertini S, Lister TR, Lombardi S, Metcalfe R, Pearce JM, Smith KL, Steven MD, Thatcher K, Ziogou F (2015). Comparison of the impacts of elevated CO_2_ soil gas concentrations on selected European terrestrial environments. International Journal of Greenhouse Gas Control.

[ref-61] Wimmer BT, Krapac IG, Locke R, Iranmanesh A (2011). Applying monitoring, verification, and accounting techniques to a real-world, enhanced oil recovery operational CO2 leak. Energy Procedia.

[ref-62] Wu Y, Ma X, Li YE, Wan YF (2014). The impacts of introduced CO_2_ flux on maize/alfalfa and soil. International Journal of Greenhouse Gas Control.

[ref-63] Xavier M, Lukasz LS, Martini X, Stelinski LL (2017). Drought stress affects response of phytopathogen vectors and their parasitoids to infection- and damage-induced plant volatile cues. Ecological Entomology.

[ref-64] Xu H (2006). Modification of normalised difference water index (NDWI) to enhance open water features in remotely sensed imagery. International Journal of Remote Sensing.

[ref-65] Zhang X, Ma X, Wu Y, Li Y (2015). Enhancement of farmland greenhouse gas emissions from leakage of stored CO_2_: simulation of leaked CO_2_ from CCS. Science of the Total Environment.

[ref-66] Zhang X, Ma X, Zhao Z, Wu Y, Li Y (2016). CO_2_ leakage-induced vegetation decline is primarily driven by decreased soil O_2_. Journal of Environmental Management.

[ref-67] Živčák M, Brestič M, Olšovská K, Slamka P (2008). Performance index as a sensitive indicator of water stress in Triticum aestivum L. Plant Soil Environment.

